# Access to inflatable penile prosthesis surgery as indicated by distances traveled among US men with Medicare

**DOI:** 10.1093/sexmed/qfad073

**Published:** 2024-02-10

**Authors:** Sirikan Rojanasarot, Kathryn Morris, Tristan Nicholson, Thomas Walsh

**Affiliations:** Health Economics and Market Access, Urology Division, Boston Scientific, Marlborough, MA 01752, United States; Health Economics and Market Access, Urology Division, Boston Scientific, Marlborough, MA 01752, United States; Department of Urology, University of Washington Medicine, Seattle, WA 98195, United States; Department of Urology, University of Washington Medicine, Seattle, WA 98195, United States

**Keywords:** erectile dysfunction, penile prosthesis implantation, inflatable penile prosthesis, travel burden, geographic barriers, health economics and outcomes research, care outmigration, patient access, urologist shortage

## Abstract

**Background:**

The significance of geographic barriers to receiving inflatable penile prosthesis (IPP) treatment is uncertain according to the existing medical literature.

**Aim:**

To describe the travel patterns of men with erectile dysfunction (ED) in the United States who underwent IPP surgery.

**Methods:**

This retrospective cohort study utilized data from the 100% Medicare Standard Analytical Files. Men aged ≥65 years with an ED diagnosis who underwent IPP surgery between January 2016 and December 2021 were identified from the database. Federal Information Processing Series codes from the National Bureau of Economic Research’s County Distance Database were used to determine geographic distances from patients’ homes to the facilities at which surgery was performed.

**Outcomes:**

Evaluations included the proportions of men who traveled outside their county of residence or state for IPP treatment and the average distances in miles traveled.

**Results:**

Among 15 954 men with ED undergoing IPP treatment, 56.4% received care out of their county for IPP, at a mean distance of 125.6 miles (range, 3.8-4935.0). Although patients aged ≥80 years were less likely to travel outside their county as compared with men aged 65 to 69 years (48.1% vs 57.1%, *P* < .001), if they traveled, they were likely to travel farther (mean, 171.8 vs 117.7 miles; *P* < .001). South Dakota had the highest proportion of men traveling outside their county for IPP treatment (91.3%; mean, 514.2 miles), while Vermont had the highest proportion traveling outside their home state (73.7%).

**Clinical Implications:**

By unveiling disparities in access, this study will potentially lead to tailored interventions that enhance patient care and health outcomes.

**Strengths and Limitations:**

Strengths include the uniqueness in (1) evaluating the proportions of patients who travel out of their county of residence or home state for IPP treatment and (2) quantifying the average distances that patients traveled. An additional strength is the large sample size due to the retrospective design and database used. The analysis did not capture all Medicare enrollees; however, it did encompass all traditional Medicare enrollees, representing approximately half of all men in the US aged ≥65 years. Limitations include not being generalizable to entire population of the US, as the study examined only Medicare enrollees. In addition, the study period includes the pandemic, which could have affected travel patterns. Furthermore, the coding and accuracy of the data are limitations of using administrative claims data for research.

**Conclusion:**

Study findings showed that many men with Medicare and ED traveled from their home geographic location for IPP treatment.

## Introduction

The prevalence of erectile dysfunction (ED) in the United States increased by 116% between 2009 and 2017 among commercially insured men,^1^ increasing the demand for ED-related urologic care. While access to ED management therapies (eg, ED-related pharmaceuticals, vacuum pumps, injections) is widespread in the United States,^2^^,^^3^ access to definitive ED therapies, such as inflatable penile prosthesis (IPP) and other therapies that can restore sexual function, may be limited due to the undersupply of urologic professionals capable of providing this level of care.^4^^,^^5^ Only 15% of urology training programs have a dedicated prosthetic urologist.^4^

The 2020 American Urological Association survey highlighted a shortage of US urologists trained in IPP and other restorative therapies.^5^ This, combined with the uneven distribution of these specialists,^5^ may limit access to IPP treatment for men with ED. Such limited access could lead to burdensome travel expenses for individuals needing to seek treatment far from their local area, possibly outside their county or state. Alternatively, some patients may forego care,^6^ leading to greatly diminished quality of life^7^ and loss of productivity.^8^^,^^9^ Geographic barriers to health care access may have a greater impact on disadvantaged and marginalized populations.^10^^,^^11^

The significance of geographic barriers to receiving IPP treatment is uncertain according to the existing medical literature. Understanding patient travel patterns and geographic areas in the country that have deficits in IPP care is important for implanters treating patients from different geographic regions or implanters who are leading men’s health fellowship programs. This study sought to gain a greater understanding of the burden of travel among patients in the United States with Medicare who received IPP treatment. The objectives were to evaluate (1) the proportion of patients who traveled out of their county or state of residence to receive IPP treatment and (2) the distance traveled to obtain IPP treatment among those who traveled out of their county or state of residence.

## Methods

### Data source

This retrospective cohort study analyzed historical data from the 100% Medicare Standard Analytical Files, which cover all traditional Medicare (Parts A and B) subscribers’ claims between January 1, 2016, and October 31, 2021. In the United States, Medicare is a federal health insurance program that primarily provides coverage for individuals aged ≥65 years. Medicare is divided into different parts, such as Part A (hospital insurance), Part B (medical insurance), Part C (Medicare Advantage plans), and Part D (prescription drug coverage), each offering specific types of coverage.^12^ It is important to note that this analysis did not include data from Medicare Advantage (Part C) enrollees. Medicare Advantage requires enrollees to obtain care only through in-network providers, while traditional Medicare allows enrollees to receive care from any Medicare provider nationwide.

### Study population

The analysis included men with Medicare who met the following selection criteria: age ≥65 years at the time of the index IPP procedure, ≥1 ED medical diagnosis claim, and IPP surgery between January 2016 and October 2021. The diagnosis of ED in the study was determined by specific clinical codes from *ICD-10-CM* (F52.xx).

### Patient demographics and variables of interest

Patients’ geographic regions were evaluated per the US Census categories: West, South, Midwest, and Northeast. Age was grouped by 5-year increments until the age of 80 years: 65 to 69, 70 to 74, 75 to 79, and ≥80. Race was categorized as American Indian, Asian, Black, White, Hispanic, other, and unknown; however, we decided to combine American Indian (0.4%), Asian (1.1%), Hispanic (4.3%), other (1.4%), and unknown (2.7%) into an “other” grouping due to the low patient counts in these categories. Eleven Charlson Comorbidity Index^13–15^ comorbidities were used to assign patients baseline comorbidity scores, which ranged from 0 to 11.

### Study outcomes

The primary study outcomes were travel patterns among men receiving IPP care. The travel patterns were defined as the proportions of patients who traveled outside their county and the proportions of patients who traveled outside their state for IPP treatment, as well as the distance that patients traveled to obtain IPP surgery. These distances were determined by Federal Information Processing Series codes,^16^ whereby a code was assigned to each patient’s county of residence and to the county where that patient underwent IPP surgery. The distance between these locations was calculated per the National Bureau of Economic Research’s County Distance Database.^17^

### Statistical analysis

Descriptive analyses were performed of the study population’s baseline characteristics, the proportions of patients traveling out of county and state, and the travel distances of patients who underwent IPP surgery. States with the highest percentage of patients who traveled out of their county and out of their state were also assessed. Tertile analyses were conducted to better understand the characteristics of patients who did and did not travel various distances outside their county for IPP treatment. Patients were categorized into 3 travel distance distribution ranges. Tertile 1 included individuals who remained in their county (0 miles traveled); tertile 2, those who traveled outside their county (3.8-33 miles); and tertile 3, all those who traveled outside their county (>33 miles). Since more than a third of the sample population did not travel outside their county to have the procedure done, the breakdown for each tertile did not equal 33%. Chi-square tests were used to assess the statistical differences in categorical variables. Sample selection and creation of analytic variables were performed with the Instant Health Data software (Panalgo). Statistical analyses were conducted with SAS (version 9.4; SAS Institute). Python was used for the creation of the Sankey diagram. Stata software (version 17; StataCorp) was used for the tertile analyses.

## Results

### Baseline characteristics

A total of 15 954 patients met the study selection criteria (Table 1). Almost half (47.7%) of these eligible patients were aged 65 to 69 years, and <5% (4.8%) were aged ≥80 years. More than half of the study population (53.6%) lived in the South region of the United States and 17.4% in the West, 16.8% in the Midwest, and 12.2% in the Northeast. Approximately three-quarters of the patients (77.2%) were White, 13.9% were Black, and 8.8% were classified as other. More than half of the patients (54.5%) had a Charlson Comorbidity Index score of 0, while 32.0% had a score ≥2. The most prevalent comorbidities were hypertension (73.7%), dyslipidemia (54.2%), and diabetes (34.1%).

**Table 1:** Patient characteristics overall and by tertile distance of travel out of their county of residence for IPP treatment.

		**No. of Patients**			
**Patient characteristics**	**Overall**	**Tertile 1 (0 miles)**	**Tertile 2 (3.8-33.0 miles)**	**Tertile 3 (>33.0-4934.0 miles)**	***P* value** ^**a**^
Sample size	15 954	6598	3678	5318	
Age, y					<.001
65-69	7611 (47.7%)	3269	1770	2572	
70-74	5193 (32.6%)	2197	1238	1758	
75-79	2378 (14.9%)	1091	524	763	
≥80	772 (4.8%)	401	146	225	
Geographic region					<.001
Midwest	2677 (16.8%)	1005	755	917	
Northeast	1942 (12.2%)	818	654	470	
South	8537 (53.6%)	3446	2143	2948	
West	2774 (17.4%)	1686	117	971	
Race					<.001
White	12 321 (77.2%)	4955	2853	4513	
Black	2221 (13.9%)	1172	592	457	
Other	1412 (8.8%)	831	233	348	
CCI score					.091
0	8693 (54.5%)	3851	1972	2870	
1	2154 (13.5%)	956	507	691	
≥2	5170 (32.0%)	2151	1199	1757	
Comorbidities					
BPH	3657 (22.9%)	1676	902	1079	<.001
CVD	4300 (26.9%)	1812	1052	1436	.018
Depression	1492 (9.3%)	641	352	499	.830
Diabetes	5437 (34.1%)	2459	1307	1671	<.001
Dyslipidemia	8653 (54.2%)	3753	2089	2811	.001
Hypertension	11 765 (73.7%)	5195	2736	3834	.004
Obesity	2487 (15.6%)	1047	585	855	.247
PVD	1531 (9.6%)	657	377	497	.303
Prostate cancer	2498 (15.7%)	974	556	968	<.001

Abbreviations: BPH, benign prostatic hyperplasia; CCI, Charlson Comorbidity Index; CVD, cardiovascular disease; IPP, inflatable penile prosthesis; PVD, peripheral arterial disease.

a Chi-square evaluated statistically significant differences at *P* < .05.

### Travel for IPP treatment

More than half of the study population (56.4%) traveled out of their county of residence for IPP surgery (Table 2). Among those who traveled outside their county for IPP surgery, the mean (SD) distance traveled was 125.6 (308.4) miles, the median distance was 39.4 miles, and the range was 3.8 to 4935.0 miles. A smaller proportion of patients aged ≥80 years (48.1%) traveled out of their county of residence for IPP surgery as compared with patients aged 65 to 69 years (57.1%), 70 to 74 (57.7%), or 75 to 79 (54.1%; chi-square, *P* < .05). However, when patients aged ≥80 years traveled out of their county of residence for IPP surgery, the mean distance traveled was farther when compared with patients in the lower age categories: 171.8 miles (455.7) vs 117.7 (277.2) for 65 to 69 years, 130.1 (325.2) for 70 to 74 years, and 135.4 (314.4) for 75 to 79 years. When the data were examined by tertiles of travel distance, the distribution of patients by age grouping did not differ between patients traveling 3.8 to 33.0 miles and >33.0 to 4934.0 miles.

**Table 2:** Characteristics and travel distances among patients who traveled out of their county of residence for IPP treatment.

		**Traveled out of, %** ^**a**^		**Distance traveled out of county, miles** ^**b**^				
**Patient characteristic**	**No. of patients** ^**c**^	**County (*n* = 8996)**	**State (*n* = 1868)**	**Mean**	**Median**	**SD**	**Min**	**Max**
Sample size	15 954	56.4	11.7	125.6	39.4	308.4	3.8	4935.0
Age, y^a^								
65-69	4342	57.1	12.0	117.7	39.4	277.2	3.8	4934.9
70-74	2996	57.7	11.7	130.1	38.9	325.2	3.8	4858.1
75-79	1287	54.1	11.6	135.4	39.4	314.4	4.3	3186.5
≥80	371	48.1	9.1	171.8	40.5	455.7	4.0	4911.7
Geographic region^a^								
Midwest	1672	62.5	15.2	124.5	37.8	268.7	10.4	1907.2
Northeast	1124	57.9	15.8	89.8	29.3	261.4	6.1	2619.4
South	5091	59.6	10.5	102.2	37.4	222.5	3.8	2298.8
West	1088	39.2	8.9	272.9	75.4	578.7	15.8	4934.9
Race^a^								
White	7366	59.8	12.6	131.9	40.7	311.8	3.8	4934.9
Black	1049	47.2	8.3	73.5	30.2	192.7	6.1	2373.3
Other	581	41.2	9.4	154.7	40.3	408.8	4.0	4858.1
CCI score^a^								
0	4842	55.7	11.3	135.0	39.1	336.6	3.8	4934.9
1	1198	55.6	10.6	104.6	37.7	244.0	3.8	2400.3
≥2	2956	57.8	12.8	121.6	40.2	281.7	3.8	3186.5
Comorbidities								
BPH^a^	1981	54.2	19.2	106.6	35.6	269.5	3.8	2561.2
CVD^a^	2488	57.9	27.1	118.5	37.9	288.3	3.8	4911.7
Depression	851	57.0	9.2	114.7	38.1	271.8	3.8	2619.4
Diabetes^a^	2978	54.8	10.8	107.6	36.5	263.4	3.8	2700.4
Dyslipidemia	4900	56.6	11.5	118.8	37.9	278.7	3.8	3186.5
ED^a^	8407	56.6	12.0	129.1	39.6	312.6	3.8	4934.9
Hypertension^a^	6570	55.8	11.1	119.7	38.7	290.4	3.8	4858.1
Obesity	1440	57.9	11.2	126.7	39.4	315.2	3.8	4911.7
PVD	874	57.1	10.1	96.0	37.6	233.1	5.5	2531.5
Prostate cancer^a^	1524	61.0	15.0	128.8	43.6	281.8	6.1	3186.5

Abbreviations: BPH, benign prostatic hyperplasia; CCI, Charlson Comorbidity Index; CVD, cardiovascular disease; ED, erectile dysfunction; IPP, inflatable penile prosthesis; PVD, peripheral arterial disease.

a Chi-square determined statistically significant differences at *P* < .05.

b Among those who traveled out of county.

c From the 15,954 included patients in the analysis, this table presents results for individuals who traveled outside their county. The total of patient subgroups in each category may not sum up to 8,996 as some patients had missing characteristic information.

A smaller proportion of patients from the West geographic region traveled out of their county of residence to receive IPP treatment as compared with patients in the other regions: 39.2% vs 62.5% for Midwest, 59.6% for South, and 57.9% for Northeast. Similar to patients aged ≥80 years, when patients from the West traveled out of their county of residence for IPP surgery, the mean (SD) distance traveled was greater than that among patients in other regions: 272.9 (578.7) miles vs 89.8 (261.4) for Northeast, 102.2 (222.5) for South, and 124.5 (268.7) for Midwest. A larger proportion of patients who identified as White traveled out of their county of residence (59.8%) vs patients who identified as Black or other race (47.2% or 41.2%, respectively). Patients who identified as Black who traveled out of their county of residence traveled a shorter mean (SD) distance to receive IPP treatment: 73.5 (192.7) miles as compared with 131.9 (311.8) for White and 154.7 (408.8) for other race. Tertile travel distance analyses showed similar findings, with patients from the West traveling greater distances and patients identifying as Black traveling shorter distances. The proportions of patients who traveled out of their county of residence and the distances traveled did not differ by Charlson Comorbidity Index scores or by the specific comorbidities evaluated.

**Figure 1:**
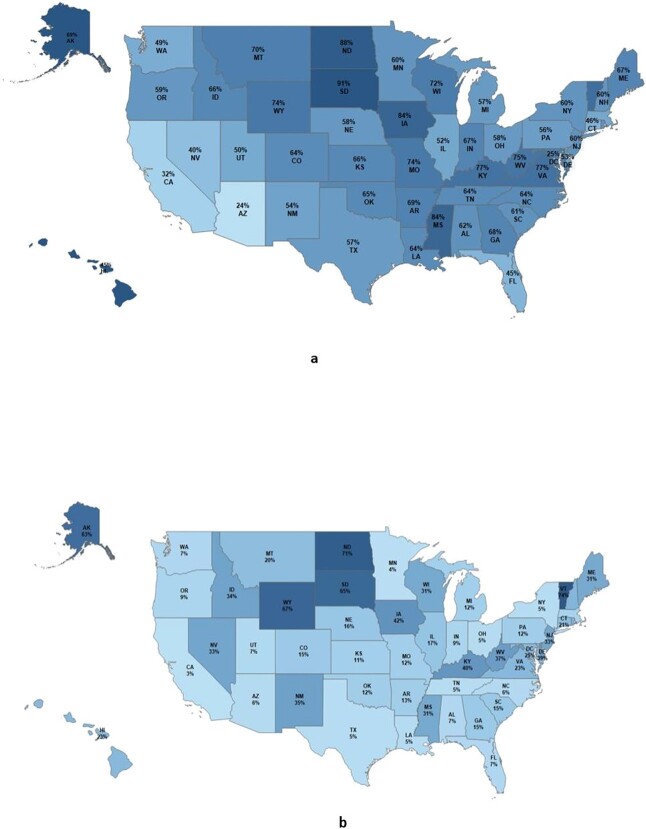
Proportion of patients who traveled (a) outside their county of residence and (b) outside their state of residence for IPP treatment by state. IPP, inflatable penile prosthesis.

Figure 1a presents the proportions of patients who traveled out of their county of residence for IPP surgery by state. The data showed that the proportions who traveled out of their county of residence for IPP surgery varied greatly by state, ranging from 91.3% in South Dakota to 24.5% in Arizona. In addition to South Dakota, states with the highest proportions of patients who traveled out of their county of residence for IPP surgery were North Dakota (88.2%), Iowa (84.3%), Mississippi (83.8%), and Vermont (78.9%).

The proportion of patients who traveled out of their state of residence for IPP surgery was 11.7% (Table 2). Among patients who traveled out of their state of residence for IPP surgery, the mean (SD) distance traveled was 410.1 (580.9) miles, the median distance was 109.2 miles, and the range was 6.5 to 4935.0 miles. The proportions of patients who traveled out of their home state for IPP surgery also varied greatly by state, ranging from 73.7% in Vermont to 3.1% in California (Figure 1b). In addition to Vermont, other states with the highest rates of travel out of state were North Dakota (70.6%), Wyoming (66.7%), South Dakota (65.2%), and Alaska (62.5%).

**Figure 2:**
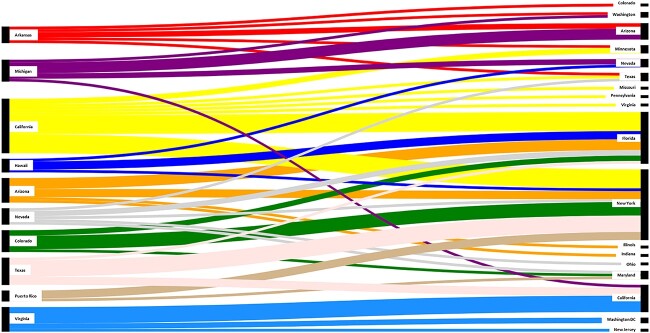
Sankey diagram summarizes the travel patterns from the top 10 states with the highest number of patients. Colors represent the states from which the patients originated (eg, Arkansas red, Michigan purple, California yellow). The right side shows where patients ended up, with all patients traveling to the state of destination represented under the state name. For example, Florida was the state most commonly traveled to, with many patients from California (yellow) and Arizona (orange) coming to Florida for IPP treatment. Many patients also traveled to New York for IPP treatment, particularly patients from Texas, Colorado, and Puerto Rico. IPP, inflatable penile prosthesis.

Figure 2 presents a Sankey diagram summarizing travel patterns from the top 10 states with the highest number of patients with IPP. The findings illustrate the diverse travel locations where patients sought IPP treatment. Overall, New York and Florida were primary destinations for IPP treatment among the patients.

## Discussion

To our knowledge, this is the first study to evaluate the distance traveled among patients with ED who received IPP treatment. This analysis of nearly 16 000 US men with Medicare insurance and ED found that more than half traveled outside their county of residence and >10% outside their home state for IPP treatment. The mean distance traveled outside the county was 125.6 miles each way, which is the equivalent for a round trip of 251.2 miles. Travel distances varied widely, with South Dakota having the highest proportion of men traveling outside their county of residence for IPP surgery (91.3%; mean distance, 514.2 miles) and Vermont having the highest proportion of men traveling outside their home state (73.7%). Overall, the study findings showed that many men traveled great distances from their geographic location for IPP treatment, thereby potentially experiencing a geographic burden in accessing the therapy.

Given the extent to which patients in this study traveled for IPP treatment, the study results suggest that there may be an undersupply of urologists who can perform IPP surgery. Understanding travel patterns and geographic areas in a country that has an IPP care deficit is critical for urologists who perform IPP surgery and treat patients from different geographic regions or for urologists who perform IPP surgery and lead men’s health fellowship programs. In a recent geographic analysis of outpatient-based IPP procedures,^18^ the authors determined that Florida (11.2%), California (7.8%), Texas (7.5%), New York (5.9%), and Michigan (4.2%) had the highest number of implanters per state. These findings align directly with the states having the lowest out-of-state migration rates for IPP procedures as well as the crude numbers of men undergoing IPP found in the current study (Figure 1b). According to the American Urological Association, only 4 out of 100 practicing urologists in the United States are currently trained to perform IPP. The same urologist workforce report emphasized that there is an insufficient supply of urologists overall in the United States and they are not optimally geographically distributed.^5^ This lack of access to urologists may result in patients foregoing urologic and ED care, leading to worsening patient outcomes and quality of life.^7^^,^^8^^,^^19–21^ There is also a need for more support for urologic training in ED and sexual health. Only 15% of urology training programs have a dedicated prosthetic urologist available.^4^ Ensuring sufficient urologists and penile prosthesis implanters could mitigate the potential physical, emotional, and social burden of untreated ED among appropriately selected patients.

Another aspect to consider is that the geographic barriers for IPP treatment may have prevented or deterred some patients from receiving IPP surgery. Geographic barriers are often cited as barriers to health care access,^6^ as they lead to rescheduled or missed appointments, delayed care, and missed or delayed treatment.^11^ Evidence has shown that patients who do not obtain medical care because of geographic or transportation barriers are more likely to be female, poorer, older, less educated, and a minority population.^10^^,^^11^^,^^22^ The delayed or missed appointments and treatments resulting from geographic or transportation barriers may lead to poorer disease management and thus poorer health outcomes.^6^ In our study, when compared with White patients, a smaller proportion of patients who identified as Black traveled out of their county of residence for IPP treatment, and those who did so traveled a shorter mean distance to receive IPP treatment. These findings may indicate that some patients who were Black did not receive IPP treatment due to transportation barriers; however, we are not able to know this with certainty, as our study did not look at those patients who wanted IPP treatment but did not receive it.

There is a rapidly increasing demand for physicians in the United States; physician shortages currently exist in many states across the nation and will likely increase over the next 10 years.^23^ The United States currently faces a lack of urologists with relevant IPP training willing and able to offer IPP and other restorative therapies.^5^ Structural change and advocacy are needed to maximize the available urology workforce, particularly since the total number of practicing urologists per capita is projected to decrease in the coming decades.^24^ The information derived from this study can inform health workforce planners, employers, educators, and policy makers regarding the development of strategies to reduce IPP implanter shortages.^23^ Our findings also provide existing implanters and fellows with a better understanding of the potential geographic areas where patient access to IPP may need improvement and where there are opportunities after the fellows graduate.

This study sheds light on the potential burden faced by patients in obtaining treatment for IPP and provides policy implications. The study found, on average, that patients undergoing IPP traveled 125.6 miles each way for the implant, equivalent to a round trip of 251.2 miles. However, previous research has indicated that IPP is typically considered a last resort for ED treatment, implying that patients may have traveled an average of 251.2 miles multiple times before having the opportunity to receive IPP care. The travel burden reported in this study could be considered as conservative as it does not capture potential additional travel distances and time for patients if they need to travel back to the same facility area for post-operative care, follow-up and teaching visits for IPP usage. Alongside the travel burden, patients who are actively employed may have had to miss work to seek ED care, resulting in a financial burden and the cost of missed opportunities for their employers. Given that the exclusion of ED treatments from coverage is a prevalent policy concern among patients with employer-sponsored health insurance, the findings of this study could offer a fresh perspective on patient care provision models. These models could maximize the clinical benefits for patients, reduce the time to definitive ED treatment, and provide cost-efficient solutions for health care sponsors and employers.

This study found that, as compared with patients in lower age categories, a smaller proportion of patients aged ≥80 years traveled out of their county of residence. However, the distance that these patients traveled was farther. A plausible explanation for the observed travel patterns among older patients could be the necessity to seek IPP care from centers of excellence or highly experienced implanters, who may not be readily available within their local vicinity.

Patients who identified as Black were less likely than White patients to travel out of their county of residence, and they traveled shorter distances for IPP treatment. Greater proportions of patients in South Dakota, North Dakota, Iowa, Mississippi, and Vermont traveled out of their county of residence for IPP surgery, and greater proportions of patients in Vermont, North Dakota, Wyoming, South Dakota, and Alaska traveled out of their home state for IPP surgery. Hence, our study findings may indicate that older, minority, and rural patients may not be obtaining IPP treatment due to the geographic barriers that they experience.

Strengths of the current study include its uniqueness in (1) evaluating the proportions of patients who travel out of their county of residence or home state for IPP treatment and (2) quantifying the average distances that patients traveled. The study used a retrospective administrative claims database, enabling efficient analysis of large numbers of patients receiving IPP treatment. Given the population identified who use Medicare, the findings are representative of men across the United States who are >65 years of age and have Medicare health insurance. While this analysis did not capture all Medicare enrollees, it did encompass all traditional Medicare enrollees, representing approximately half of all men in the United States aged ≥65 years.^25^

The limitations of the study mostly relate to its retrospective and observational design, which makes it difficult to capture all clinically relevant variables, ensure the accuracy of the data, and draw causal inferences. Retrospective database analyses rely on coding accuracy; however, there may be coding errors and misclassifications, as well as inaccurate, incomplete, or missing codes that lead to under- or overreporting of outcomes. Furthermore, this is a retrospective study, which makes it difficult to determine why patients chose to seek IPP surgery where they did, with some traveling great distances. Additionally, this study included some patients treated during the COVID-19 pandemic, which likely affected travel patterns. Many elective procedures (including IPP services) were severely curtailed during the first year of the pandemic to minimize possible COVID-19 exposure and redirect staff to COVID-19–related care,^26^^,^^27^ which may have forced patients to delay IPP treatment or travel greater distances to receive IPP treatment. Finally, the findings from this database study may not be generalizable to all populations of patients with ED in the United States given that the study population does not include Medicare Advantage, other managed care enrollees, or individuals with other types of insurance. For example, managed care plans often limit the greatest coverage to in-network providers, and patients may have needed to travel to find eligible providers who could provide IPP treatment.

## Conclusion

This study provides evidence that many men with Medicare insurance and ED who receive IPP treatment travel great distances to receive surgery, suggesting that there may be geographic and/or transportation barriers to accessing IPP treatment for many US Medicare patients. As this research did not assess the reasons why patients may have opted to travel for IPP treatment, future research could explore whether travel is related to an insufficient supply of urology specialists trained in and available to provide IPP treatment. Future research may also evaluate the extent to which geographic barriers may have prevented or deterred minority or vulnerable populations from receiving timely IPP treatment. As insufficient access to definitive ED care can lead to diminished quality of life, addressing gaps and disparities in access to care should be a priority in ensuring adequate urologic care for men with Medicare insurance.
